# Impact of charge transport on current–voltage characteristics and power-conversion efficiency of organic solar cells

**DOI:** 10.1038/ncomms7951

**Published:** 2015-04-24

**Authors:** Uli Würfel, Dieter Neher, Annika Spies, Steve Albrecht

**Affiliations:** 1 Department of Dye and Organic Solar Cells, Fraunhofer Institute for Solar Energy Systems ISE, Heidenhofstrasse 2, 79100 Freiburg, Germany; 2 Material Research Centre FMF, University of Freiburg, 79104 Freiburg, Germany; 3 Institute of Physics and Astronomy, University of Potsdam, 14476 Potsdam-Golm, Germany

## Abstract

This work elucidates the impact of charge transport on the photovoltaic properties of organic solar cells. Here we show that the analysis of current–voltage curves of organic solar cells under illumination with the Shockley equation results in values for ideality factor, photocurrent and parallel resistance, which lack physical meaning. Drift-diffusion simulations for a wide range of charge-carrier mobilities and illumination intensities reveal significant carrier accumulation caused by poor transport properties, which is not included in the Shockley equation. As a consequence, the separation of the quasi Fermi levels in the organic photoactive layer (internal voltage) differs substantially from the external voltage for almost all conditions. We present a new analytical model, which considers carrier transport explicitly. The model shows excellent agreement with full drift-diffusion simulations over a wide range of mobilities and illumination intensities, making it suitable for realistic efficiency predictions for organic solar cells.

## Abstract (web summary)

Fitting current–voltage curves of organic solar cells with the Shockley equation often results in artificially high ideality factors. Here, the authors analyse inadequacy of the equation and propose an analytic model, which allows prediction of the efficiency potentials by explicit consideration of charge-carrier mobilities.

## Graphical abstract

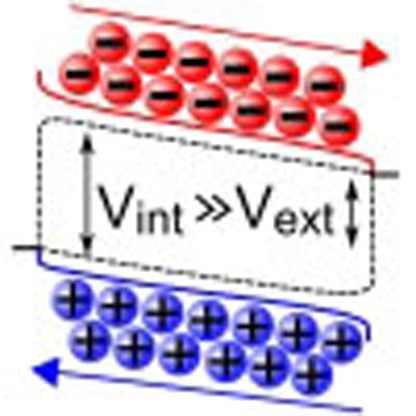

With the development of new photoactive materials and the optimization of device architectures, the performance of organic solar cells has significantly increased over the last 5 years. However, their power-conversion efficiency (PCE) remains well below the Shockley–Queisser limit^1^. This raises the question about the physical processes limiting the efficiency. In the past, several models have been put forward, mainly considering losses due to geminate recombination^2^,^3^, limiting the short-circuit current *J*_SC_, or due to non-radiative recombination pathways reducing mainly the fill factor (FF) and the open-circuit voltage *V*_OC_ (refs 4, 5). Recent experimental and theoretical work, however, led to conclude that the performance of different polymer:fullerene blends to be largely affected by inefficient charge extraction due to low mobilities, in particular in systems with effective charge generation and/or large active layer thickness^6^,^7^,^8^.

To describe the current–voltage characteristics (*JV* curve) of a solar cell quantitatively by means of an analytical expression, researchers often use the Shockley equations^4^,^9^,^10^,^11^,^12^,^13^,^14^,^15^,^16^:


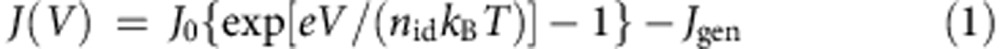


with *J*_0_ being the dark generation (or saturation) current density, *e* the elementary charge, *V* the potential difference between the contacts, *k*_B_ Boltzmann's constant and *T* the temperature. In equation (1), the total current density *J* is the sum of the current density of photogenerated charge carriers *J*_gen_ and the recombination current density *J*_rec_(*V*)=*J*_0_ exp[*eV*/(*n*_id_*k*_B_*T*)]. In the latter term, *n*_id_ is the ideality factor, which depends on the exact recombination mechanism and hence on the reaction order. The recombination rate *R* is a function of the density of (free) electrons and holes, *n*_e_ and *n*_h_, respectively, which themselves depend on the positions of the quasi Fermi levels. The quasi Fermi level of the electrons in the electron transport level (ETL, corresponding to the lowest unoccupied molecular orbital (LUMO) of the acceptor) is denoted as *E*_FE_ and the quasi Fermi level of the electrons in the hole transport level (HTL, corresponding to the highest occupied molecular orbital (HOMO) of the donor) is denoted as *E*_FH_. The recombination rate *R* for a particular recombination process can generally be written as:


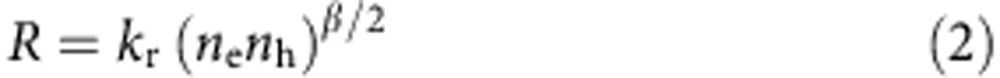


with *k*_r_ being the recombination coefficient and *β* the reaction order of the recombination process. With equation (2), the recombination current density can be expressed as:


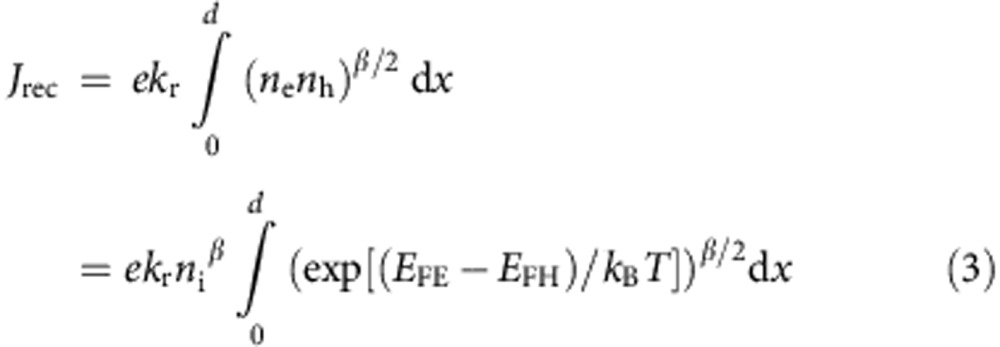


where *n*_i_ is the intrinsic charge-carrier density, *d* the thickness of the photoactive layer and *J*_0_=*ek*_r_*n*_i_^*β*^ *d* is the corresponding dark-generation current density.

For infinitely large electron and hole conductivities, the quasi Fermi levels (and with that the quasi Fermi level splitting) are constant throughout the active layer. In addition, for properly chosen contacts, *E*_FE_ (*E*_FH_) aligns with the Fermi level of the electron (hole) contact, resulting in:


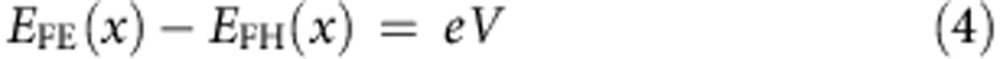


at any position *x* within the photoactive layer. In this case, equation (3) becomes *J*_rec_=*J*_0_ exp [*β*(*E*_FE_−*E*_FH_)/2*k*_B_*T*]. The bias across the active layer can be further related to the applied voltage by *V*=*V*_ext_−*R*_S_*J*, with *R*_s_ being an external (constant) series resistance. Applying the assumption of infinite conductivities of equation (4) in equation (3) gives the Shockley equation (equation (1)) and a comparison with equation (3) delivers the correlation of the ideality factor with the reaction order:


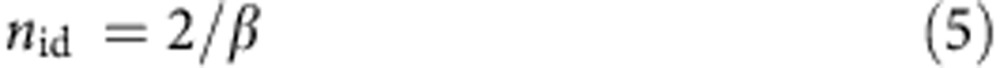


For direct recombination, the rate is given by *R*=*k*_dir_*n*_e_*n*_h_, with *k*_dir_ being the coefficient for direct recombination, and the ideality factor *n*_id_=1 in this case. For Shockley–Read–Hall recombination^17^, it is *n*_id_=2 and for Auger recombination^18^ *n*_id_=2/3.

As transport was not considered in the derivation of equation (1), it can be regarded as an idealization that describes a device with infinitely large conductivities (and hence mobilities) of electrons and holes. When Shockley and co-workers^9^ derived their expression this was a reasonable assumption, as their aim was to describe the behaviour of diodes. By that time, diodes were solid-state devices comprising *pn*-junctions made from highly crystalline inorganic materials with rather high mobilities such as, for example, Ge, Si or GaAs.

In contrast to crystalline inorganic semiconductors, organic semiconductors display quite low mobilities^19^,^20^. Hence, the basic assumption of equation (4) is not fulfilled for organic solar cells.

To investigate the effect of limited transport properties, numerical simulations were performed with the semiconductor device simulation tool TCAD Sentaurus from Synopsys Inc.^21^. The model is based on mimicking the photoactive layer by a one-dimensional effective semiconductor with the ETL corresponding to the LUMO of the acceptor phase and the HTL to the HOMO of the donor phase^22^. The electrodes are defined by their work functions and the surface recombination velocities of electrons and holes. As state-of-the-art organic solar cells employ thin interlayers from, for example, TiO_x_, ZnO, MoO_3_, NiO and V_2_O_5_ to enhance charge-carrier selectivity of the contacts^23^,^24^,^25^,^26^, electron- and hole-selective layers were included into the one-dimensional stack. For the electron-selective layer the ETL is aligned to the ETL of the photoactive layer, whereas the holes encounter a large energetic barrier of 1.7 eV and vice versa for the hole-selective layer (Supplementary Fig. 1), thus suppressing surface recombination at the electrodes. The parameters are summarized in Table 1 and realistic values were used^27^,^28^. Intragap states were not considered; only direct (bimolecular) recombination was taken into account according to *R*=*k*_dir_*n*_e_*n*_h_.

For sake of simplicity, the generation rate was restricted to the photoactive layer and assumed to be spatially homogeneous. Only balanced mobilities (*μ*_e_=*μ*_h_=*μ*) were considered and series or parallel resistances were not regarded in the simulations (*R*_S_=1/*R*_P_≡0). Here we analyse the effect of charge-carrier transport on the current–voltage characteristics and PCE of single-junction photovoltaic devices. We demonstrate that it is meaningless to apply the Shockley equation to current–voltage curves under illumination and to extract information on physical parameters such as the recombination order or the apparent shunt resistance *R*_p_. Further, we propose a new analytical approach, which is suited to accurately reproduce simulated current–voltage characteristics for a wide range of parameters and thus to predict achievable PCEs.

## Results

### Drift-diffusion simulations of *JV* curves

**Figure 1:**
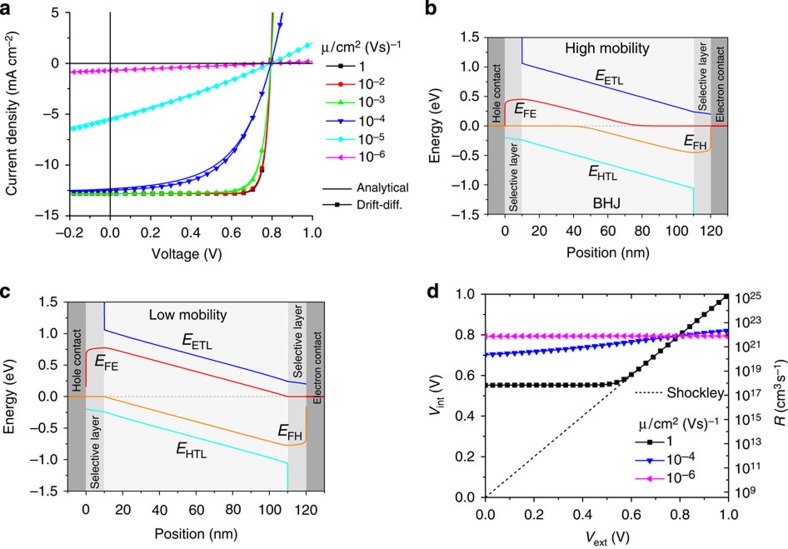
Effect of the charge-carrier mobility on the *JV* characteristics. (**a**) *JV* curves for six different charge-carrier mobilities. Thin lines with symbols show drift-diffusion simulations with the parameters in Table 1, while thick lines are the results of our analytical approximation. (**b**,**c**) show the corresponding energy diagrams under short-circuit conditions (*V*_ext_=0 V) for *μ*=1 and 10^−6^ cm^2^(Vs)^−1^, respectively. (**d**) Internal voltage *V*_int_ (left *y* axis) versus external voltage *V*_ext_ as extracted from the simulated *JV* curves with equation (6) for three different mobilities, compared with the Shockley equation (dotted line). The right *y* axis shows the corresponding recombination rate *R*. All graphs correspond to an illumination intensity of ‘1 sun'.

The simulated *JV* curves under an intensity of ‘1 sun' are plotted in Fig. 1a as thin lines with symbols. The thick solid lines are results of our analytical model, which will be explained in detail further below.

This figure illustrates the large effect of charge-carrier mobility on the shape of the *JV* curve under illumination. Lowering the mobilities first affects the FF, but *J*_SC_ is also significantly reduced for *μ*≤10^−5^ cm^2^(Vs)^−1^. In addition, the forward current density decreases strongly with lower mobilities.

These changes go along with a drastic violation of the main condition in equation (4). Figure 1b,c plot calculated band diagrams for two different mobilities under ‘1 sun' illumination at 0 V (short-circuit conditions). If the conductivities were infinitely large, no separation of the quasi Fermi levels would occur in the active layer, which is indicated by the dashed horizontal lines, hence corresponding to the assumption of the Shockley equation. In contrast, even for a high-mobility *μ*_e_=*μ*_h_=1 cm^2^(Vs)^−1^, the quasi Fermi level splitting *E*_FE_−*E*_FH_ in the bulk is considerably large and increases progressively with decreasing mobility. This proves that for all mobilities considered here, the quasi-Fermi level splitting is significantly larger than the external voltage *V*_ext_ in the voltage range 0≤*V*_ext_≤*V*_OC_. This has been confirmed recently by Schiefer *et al.*^6^,^7^ and Albrecht *et al.*^8^. The large splitting of the quasi-Fermi levels is the consequence of the accumulation of free charge carriers due to poor transport properties. As a result, non-geminate recombination is increased. This is the main cause for the continuous reduction of the FF and the short-circuit current density.

To identify a useful parameter describing the strength of recombination, we first calculated *J*_rec_ from *J*_rec_(*V*)=*J*(*V*)+*J*_gen_. Next, an internal voltage *V*_int_ is defined according to:


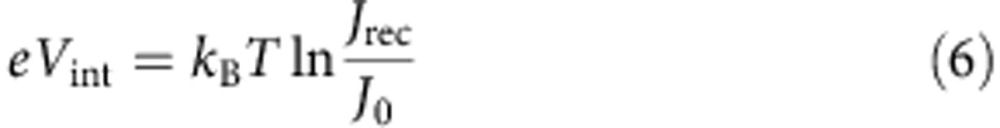


Here, *eV*_int_ is the quasi-Fermi level splitting, which, if being constant throughout the entire active layer, would cause the same *J*_rec_. Figure 1d shows *V*_int_ as a function of the external voltage *V*_ext_ under ‘1 sun' illumination for the Shockley equation (where *V*_int_≡*V*_ext_) and exemplarily for three different mobilities. For almost all conditions, *V*_int_ is significantly larger than the external bias. Therefore, an important conclusion is that for typical mobilities in organic semiconductors, the Shockley equation massively underestimates the quasi-Fermi level spitting in the bulk and thereby the carrier density. High carrier densities have been reported by several authors even at short-circuit conditions, supporting the above findings^29^,^30^. For example, for the lowest mobility *μ*=10^−6^ cm^2^(Vs)^−1^ and at *V*_ext_=0, the device is internally almost under open-circuit conditions, causing ∼95% of the photogenerated carriers to recombine. Even for *μ*=1 cm^2^(Vs)^−1^, the internal voltage *V*_int_ has a considerably high value of 552 mV under external short-circuit conditions. The above described effects are even more pronounced for increased thickness of the photoactive layer as well as for higher illumination intensities as can be seen in Supplementary Fig. 2a–e.

Based on the presented results, our simulations provide consistent proof that the condition of equation (4) is strongly violated in organic solar cells (except near OC conditions). Consequently, it is not meaningful to write the recombination current as a sole function of the bias across the active layer. We arrive at the conclusion that the Shockley equation is not applicable to organic solar cells, and that it cannot be used to analyse the current–voltage characteristics of these cells under illumination, to extract information about the recombination mechanism. This has several severe consequences. First, the difference between the current density under illumination, *J*_illu_, and in the dark, *J*_dark_, often denoted as the photocurrent *J*_ph_, has no physical meaning. It is neither equal to the overall photogenerated current nor does it mirror the recombination of only photogenerated charge. It is only *J*_illu_, which takes into account all generation processes and all recombination channels (photogenerated charge with photogenerated charge, injected with injected and photogenerated with injected). However, owing to the need of a driving force to establish *J*_illu_ within the low-mobility semiconductor bulk, the analysis of *J*_illu_(V) does not deliver information about the true ideality factor (except near *V*_OC_). In addition, the widely used extended version of the Shockley equation: 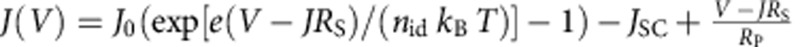 is not applicable and the often-used approach to extract the shunt resistance *R*_p_ from the slope of *J*_illu_ near short circuit is not useful. Finally, the PCE becomes not only a function of the bandgap but also of the mobility, even if the generation of charge carriers is field independent and non-geminate recombination is slow. These three issues will be considered in more detail in the following.

### Generation current and photocurrent

**Figure 2:**
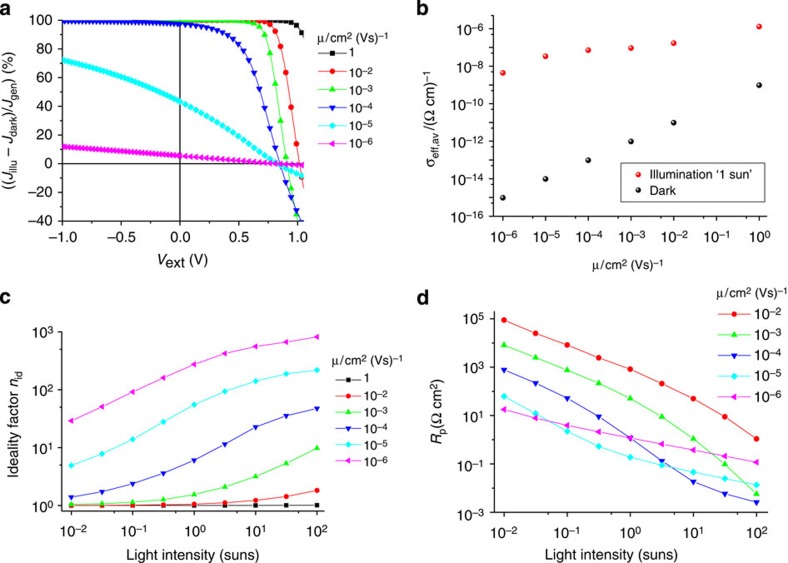
Photoconductivity and ideality factor. (**a**) Relative extraction rate of the photogenerated current as a function of the external voltage for six different mobilities at an illumination intensity of ‘1 sun'. (**b**) Corresponding effective average conductivity (see text) at *V*_ext_=0 as a function of mobility under illumination and in the dark. (**c**) Ideality factor *n*_id_ as a function of light intensity for six different mobilities. (**d**) Apparent shunt resistance *R*_p_ as the function of light intensity as determined from the inverse slope of the simulated *J*_illu_(*V*) at *V*=0. The case of *μ*=1 cm^2^(Vs)^−1^ was left out for better visualization.

As pointed out above, it is quite common to define ‘the' photocurrent, *J*_ph_ via *J*_ph_=*J*_illu_−*J*_dark_. Figure 2a plots this quantity, normalized by the generation current: (*J*_illu_−*J*_dark_)/*J*_gen_, that is, the relative extraction efficiency of the photogenerated current *J*_gen_ (which for an illumination intensity of ‘1 sun' was set to 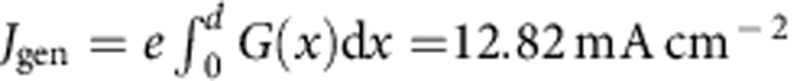). As expected, providing additional driving force by applying a negative bias voltage helps to extract more photogenerated charge carriers in those cases where the extraction at *V*_ext_=0 is incomplete due to a low mobility. However, the quantity *J*_illu_−*J*_dark_ can by no means be interpreted as the generation current, that is, *J*_gen_. This is only true for mobilities being so large that the driving forces for the transport of electrons and holes can be neglected (and if external resistances can be neglected^31^). In fact, the extraction efficiency decreases strongly with increasing forward bias and the larger the mobilities the higher the voltage where the extraction efficiency starts to decrease. It should be noted here that for an ideal diode obeying equation (1) the difference between current under illumination and current in the dark is independent of voltage and equals the generation current: *J*_illu_−*J*_dark_ ≡−*J*_gen_. The reason for the voltage dependence in forward direction is the fact that the concentration of electrons and holes for a given voltage *V*_ext_ is higher for an illuminated solar cell than for one in the dark. The additional generation of charge carriers by the light source increases their concentration and hence also their conductivity. As a consequence, a smaller part of the applied voltage *V*_ext_ is required as driving force for the transport and hence the internal voltage *V*_int_, and thus the (recombination) current is higher. This can lead (depending on the exact parameters) to the often-observed effect of intercepting dark and illuminated *JV* curves, that is, the *JV* curve under illumination ‘overtakes' the one measured in the dark. In Fig. 2a, this becomes visible by the negative values of (*J*_illu_−*J*_dark_)/*J*_gen_.

To highlight this phenomena, the spatial average of the effective charge-carrier conductivity 
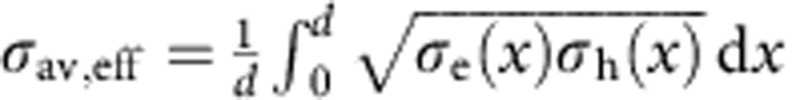
 is plotted versus the mobility at ‘1 sun' for short-circuit conditions in Fig. 2b. The conductivity of electrons and holes is given by: *σ*_e,h_=*eμ*_e,h_ × *n*_e,h_. For a given mobility, the conductivity increases strongly under illumination due to the additional generation of charge carriers. The larger conductivity then leads to larger currents in forward direction and results in the crossing of dark and illuminated *JV* curves. As expected, the increase in conductivity under illumination and short-circuit conditions is less pronounced for higher mobilities.

### Ideality factor and shunt resistance

According to the Shockley theory, for a homogeneous carrier distribution and with equation (4) being valid, the value of the ideality factor is exclusively determined by the recombination order. However, for moderate or small mobilities the relative recombination rate and hence the ideality factor will become a function of both the mobility and the illumination intensity. To illustrate this effect, *JV* curves were simulated for different mobilities and illumination intensities between 0.01 suns and 100 suns (see Supplementary Fig. 2 for exemplary curves). From these, the ideality factor was determined via:


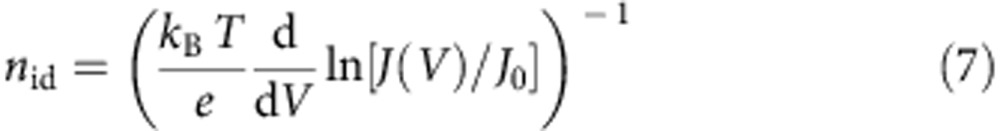


Hereby, a linear fit (of the logarithmically plotted curve) was performed in the fourth quadrant, that is, 0≤*V*≤*V*_OC_. The results are plotted in Fig. 2c, showing a very large variation of the ideality factor with charge-carrier mobility and illumination intensity. The only exception is *μ*=1 cm^2^(Vs)^−1^ where the ideality factor is unity for all light intensities. The lower the value for *μ*, the more the ideality factor deviates from 1 and the lower the intensity where *n*_id_ starts to increase. Notably, the apparent ideality factor determined via equation (7) can become very large, although direct free charge-carrier recombination was the only recombination pathway in our simulations and, therefore, *n*_id_=1 should be expected. This proves that applying the Shockley equation to the *JV* curves of low-mobility carrier devices results in ideality factors that lack real physical meaning. As shown in Fig. 2c, this violation becomes most severe for large generation currents (efficient charge generation and/or high illumination intensity) and low mobilities.

It has, indeed, become quite common to deduce information on the order of recombination and with that on the value of the ideality factor from other approaches than fitting *J*_illu_(V) curves in the fourth quadrant. For example, the bias dependence of the dark current density or of the electroluminescence intensity is analysed to determine *n*_id_^32^,^33^. However, as shown in Supplementary Fig. 3, the applicability of this approach becomes questionable for low carrier mobilities and high currents. Alternatively, the analysis can be restricted to the exponential part of dark *JV* curves, but this requires devices with very-low leakage currents^34^. A more accurate method is the determination of the ideality factor from the dependence of *V*_OC_ on the light intensity. Here, the charge-carrier densities vary over several orders of magnitude and thus more information can be extracted^35^,^36^. Although these measurements are not influenced by carrier transport, the accurate analysis of the results becomes difficult if the spatial distributions of electrons and holes are strongly asymmetric, which is the case, for example, for a small layer thickness or unintentional background doping^13^,^37^,^38^. In addition, surface recombination will alter the carrier profiles^26^ and lead to a reduction of the ideality factor.

We also find that low mobilities cause an apparent shunt resistance *R*_p_ (although 1/*R*_P_=0 in all our simulations). The concept of ‘the' shunt resistance is frequently used in the literature to account for the non-zero slope of the *J*_illu_(*V*) curve at short-circuit conditions: *R*_P_=(d*J*(*V*)/d*V*)^−1^|_*V*=0_. The analysis of experimental *JV* curves with this approach yields values for *R*_p_ of typically few tens to several thousand Ωcm^2^ (refs 39, 40, 41). Our simulations reveal that non-zero slopes of the *JV* curves are an inevitable consequence of low charge-carrier mobilities. Values for the apparent shunt resistance deduced from the simulated *JV* curves vary, indeed, over a very wide range, depending strongly on mobility and generation rate (Fig. 2d). The large discrepancy between the value of *R*_p_ used in the simulation (infinity) and the one deduced from the slope of the simulated *JV* curves with the extended Shockley equation again proves its non-applicability to organic solar cells for virtually any typical mobility and illumination intensity.

### An analytical model for *JV* curves of organic solar cells

For non-zero currents, the voltage drops related to the transport of electrons and holes through a resistive medium, that is, the photoactive layer, results in large differences between the external voltage *V*_ext_, which is applied (and which can be measured directly), and the internal voltage *V*_int_. As only the latter determines the recombination rate, the description of a *JV* curve with equation (1) using *V*_ext_ becomes worse as the charge-carrier conductivities get lower, and any approach to relate *J*_illu_ to *V*_ext_ in a trivial form will fail.

However, if we recalculate *JV* curves with the help of equation (1) but replace *V*_ext_ with *V*_int_ (*V*_ext_), the resulting curves are identical to the ones that were simulated with different mobilities. This is of course expected, as we excluded all loss mechanisms other than direct recombination between electrons and holes.

An interesting question is therefore how this internal voltage can be determined for real devices. One approach was followed by Würfel *et al.*^42^,^43^ with dye solar cells, where they managed to incorporate a third electrode into a dye solar cell, thus contacting the electron transport material, that is, the nanocrystalline TiOx, on both sides. Thus, they could show directly that under external short-circuit conditions, the internal voltage was already ∼75% of the *V*_OC_. This is in full accordance with the analysis presented here.

Although the approaches outlined above are not applicable to organic solar cells, the authors demonstrated recently two different methods to arrive at an approximate value for *V*_int_ in a working device^7^,^8^. Both relied on the assumption that the gradients of the quasi Fermi energies are constant throughout the photoactive layer, and that they are identical for electrons and holes (equation (8)). Although the assumption is not strictly fulfilled in real solar cells, it seems to be a reasonable approach as can be seen from the simulation results shown in Fig. 1b,c. We will show in the following that under this approximation, an accurate analytical description of the *JV* curves under illumination becomes feasible for a wide range of mobilities and generation currents. First, the gradient of the quasi Fermi potential is related to the current density *J*_e,h_ and the electrical conductivity *σ*_e,h_ of electrons and holes via^7^


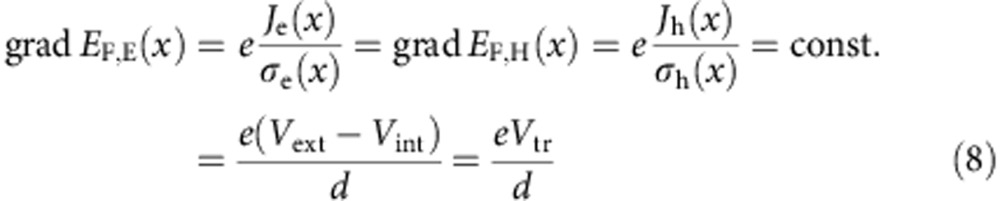


Therein, *σ*_e,h_=*eμ*_e,h_ × *n*_e,h_, with *μ*_e,h_ being the charge-carrier mobilities. *V*_tr_ is the voltage drop required for the charge-carrier transport through the active layer, which can also be expressed as *V*_tr_=*JR*_tr_. The transport resistance *R*_tr_ is a non-ohmic resistance related to the charge-carrier conductivities and the thickness of the photoactive layer. It follows from equation (8) that the internal voltage is also constant throughout the photoactive layer. In this case, the external voltage can be related to the internal voltage via


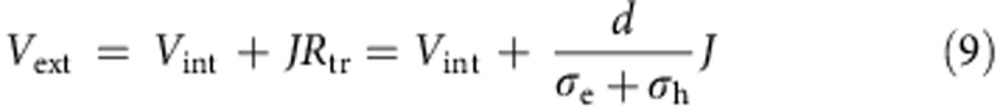


The conductivity can be further approximated by


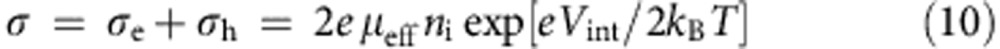


with *n*_i_ being the intrinsic carrier density and 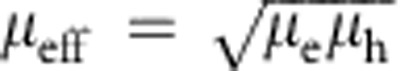 being an effective mobility (see Supplementary Note 1 for the derivation of these terms).

To analytically describe *J*(*V*_ext_), first the recombination current is calculated via


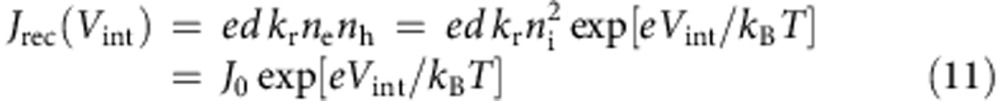


Next, the total current is obtained by adding the generated current:


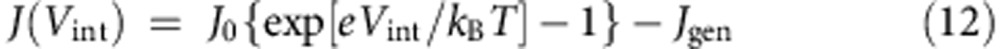


Finally, by using equations (12) and (10) for equation (9), we arrive at


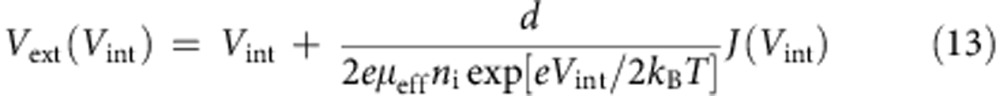


By these means, the current density and the external voltage can both be calculated from *V*_int_. As values for *μ* and *k*_r_ can however be determined separately^44^,^45^,^46^,^47^,^48^, the intrinsic charge-carrier density *n*_i_ remains as the only fitting parameter.

As a first test of the applicability of this approach, we have calculated *JV* curves with the parameters given in Table 1 and compared them with the *JV* curves from the full drift-diffusion simulations. We find excellent agreement between the simulated and the analytic approach for all mobilities as demonstrated in Fig. 1a.

We also find good agreement when applying this approach to a layer thickness of 300 nm and when changing the illumination intensity to either 0.1 suns or 10 suns (see Supplementary Fig. 2). Thus, for balanced mobilities our analytical approach shall provide an accurate analytical description of *JV* curves of organic solar cells over a wide range of mobilities and generation currents. The case of unbalanced mobilities is shown in the Supplementary Fig. 4. It can be seen that in this case our approach delivers very reasonable results as well.

### The achievable PCE

**Figure 3:**
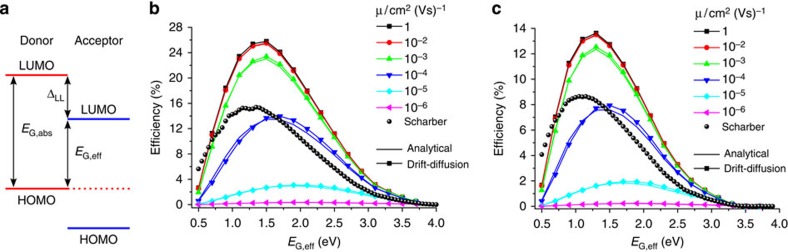
The effect of mobility on the achievable PCE. (**a**) Scheme of the two band gaps involved. (**b**) Efficiencies are plotted as a function of effective semiconductor band gap, without an offset between donor and acceptor, that is, *E*_G,abs_=*E*_G,eff_ and (**c**) with an offset *Δ*_LL_ of 0.5 eV. Shown are the results for six different mobilities calculated either via the analytical approach (thin lines with symbols) or via drift-diffusion simulations (thick lines) in comparison with the Scharber model^49^. For parameters, see text.

Finally, we consider the effect of the charge-carrier mobility on the achievable PCE. Figure 3 shows the efficiency as a function of the absorber band gap for different mobilities, either with the analytical approach and with our drift-diffusion simulations, compared with the prediction by the model of Scharber *et al.*^49^. A variable offset between the LUMO levels of donor and acceptor was used, depicted in Fig. 3a. The band gap of the donor was used to calculate the photogenerated current via 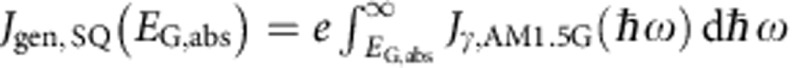 assuming full absorption of all photons of the AM1.5G spectrum with photon energies *ℏω*≥*E*_G,abs_, according to the Shockley–Queisser limit. The dark-generated current *J*_0,SQ_(*E*_G,eff_) was determined using the band gap of the effective semiconductor on the basis of the black body spectrum with *T*=300 K: 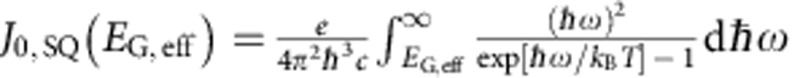, where c is the speed of light. This way, we made use of the fact that the absorption via charge transfer (states) does hardly contribute to the overall photogenerated current, while luminescence measurements show that most charge carriers do recombine via the effective semiconductor gap^4^,^50^,^51^. It is noteworthy that our study focuses on the influence of limited charge-carrier transport and the general findings presented here would not be altered even if the absorption via the effective band gap played a more significant role.

Next, an amplification factor *α* was determined as the ratio of *J*_0_ (using the parameters in Table 1) and 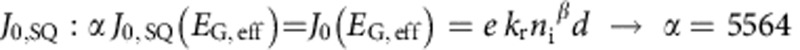. This amplification factor expresses the fact that in organic solar cells, most charge carriers do not recombine radiatively (in contrast to the Shockley–Queisser limit), but rather through additional, non-radiative pathways^4^,^51^. For simplicity, this *α*=5564 was used to calculate the dark-generated current for each effective band gap according to *J*_0_(*E*_G,eff_)=*αJ*_0,SQ_(*E*_G,eff_).

Figure 3b shows the results for equal band gaps of absorber and effective semiconductor, that is, no offset *Δ*_LL_ between the LUMO levels of donor and acceptor (and of course also between their HOMO levels for excitons generated in the acceptor), while Fig. 3c corresponds to *Δ*_LL_=0.5 eV. The results of our analytical approach are well in accordance with the full drift-diffusion simulations, meaning that the assumptions leading to equation (13) remain valid for a wide range of bandgaps and mobilities. The figures also demonstrate the large impact of the charge-carrier mobility on the device efficiency. Efficiencies beyond 25% are predicted only for zero offset, a condition that is rather unlikely to realize with organic donor–acceptor blends. Nevertheless, efficiencies of 12% and higher are within reach for a realistic offset of 0.5 eV, provided that the mobility is in excess of 10^−3^ cm^2^(Vs)^−1^.

These predictions are in good agreement to recent efficiency values. For example, Proctor *et al.*^52^ compared mobility and FF values for various solution processed small molecule-based bulk heterojunction solar cells. It is shown that balanced mobilities in excess of 2 × 10^−4^ cm^2^(Vs)^−1^ are needed to achieve high FFs (and PCEs). Notably, a record efficiency of 10.8% was achieved in blends of carefully designed polymers with fullerene^53^. These blends had exceptionally high hole mobilities of 1.5−3.0 × 10^−2^ cm^2^(Vs)^−1^. Given the fact that the external quantum efficiency (EQE) was about 0.85 on average and that *E*_G,abs_ was about 1.55 eV, efficiencies of >13% should be in reach on further optimization of the absorption properties, consistent with our predictions in Fig. 3.

For comparison, we plotted the values according to the model of Scharber *et al.*^49^ Scharber *et al.* predicted PCE for various values of *E*_G,abs_ and *E*_G,eff_, assuming a constant value of 0.65 for both EQE and FF, and assuming e*V*_OC_=*E*_G,eff_−0.3 eV. The model of Dennler *et al.*^54^ is very similar, using a value of 0.9 for the EQE and 0.7 for the FF. As both models do not consider charge-carrier transport, they are not capable to predict efficiencies as a function of mobility. A more sophisticated model was presented by Koster *et al.*^2^ The authors analysed the effect of CT absorption, reorganization energy and dielectric permittivity *ɛ* on the device performance, considering both geminate and non-geminate recombination. An important result of this work is that raising *ɛ* allows for smaller offsets *Δ*_LL_, enabling efficiencies beyond 20%. However, the effect of charge-carrier mobility is not explicitly treated in this work.

As there is no fundamental reason for *Δ*_LL_ to be at least 0.5 eV, we also determined the maximum efficiency for smaller offsets. For mobilities of 10^−2^ cm^2^(Vs)^−1^ or larger and *Δ*_LL_=0.3 eV, we find an efficiency of 18%, and for *Δ*_LL_=0.2 eV the maximum efficiency is already slightly above 20%. If the recombination coefficient *k*_r_ could be reduced by one order of magnitude, the *V*_OC_ would increase by 59.6 mV (at 300 K). As the FF is also improved, the maximum efficiency for *Δ*_LL_=0.2 eV reaches 22% for mobilities of at least 10^−2^ cm^2^(Vs)^−1^ and even for mobilities of 10^−4^ cm^2^(Vs)^−1^, it still is almost 17%.

## Discussion

We demonstrated that the Shockley equation cannot be applied to low-mobility materials such as those typically used in organic solar cells. The poor transport properties cause accumulation of charge carriers in the photoactive layer and this effect becomes more and more prominent with decreasing mobility. As a consequence, for almost all conditions encountered in organic solar cells, the separation of the quasi Fermi levels in the photoactive layer (internal voltage) differs substantially from the externally applied voltage. For this reason, there is no trivial relation between the charge-carrier concentrations in the illuminated solar cell and the external voltage, which is however an assumption of the Shockley equation. A further consequence is that parameters such as ideality factors or apparent shunt resistance determined via the simple or extended Shockley equation will result in values that lack real physical meaning. Therefore, it is not possible to extract correct information about the reaction order of the recombination process and the photogenerated current, by applying the Shockley equation to current–voltage characteristics of organic solar cells under illumination.

An analytical model is presented that explicitly considers the implication of poor charge-carrier transport. We have obtained excellent agreement of the presented analytical model with the results of full drift-diffusion simulations for a wide range of mobilities, illumination intensities and active layer thicknesses. In contrast to other models, it allows predicting efficiency potentials by explicit consideration of charge-carrier mobilities, which has been very tedious to date in an analytical way.

## Author contributions

U.W. and A.S. carried out the simulations. D.N. and U.W. developed and evaluated the analytical approach. U.W., A.S. and D.N. wrote the paper, with input from S.A.

## Additional information

**How to cite this article**: Würfel, U. *et al.* Impact of charge transport on current–voltage characteristics and power-conversion efficiency of organic solar cells. *Nat. Commun.* 6:6951 doi: 10.1038/ncomms7951 (2015).

## Supplementary Material

Supplementary Information Supplementary Figures 1-4 and Supplementary Note 1

## Figures and Tables

**Table 1:** Parameters used for the numerical simulations.

**Parameters**	**Meaning**	**Value**
*T*	Temperature	300 K
		
*Photoactive layer*		
*G*	Generation rate (varied)	8 × 10^21^ cm^−3^ s^−1^ (at ‘1 sun')
*d*_abs_	Thickness of photoactive layer	10^−5^ cm (100 nm)
*ɛ*_abs_	Dielectric permittivity of photoactive layer	3.4
*E*_G,abs_	Band gap energy of photoactive layer	1.3 eV
*N*_ETL (HTL)_	Effective density of states in the ETL (HTL)	5 × 10^20^ cm^−3^
*k*_dir_	Recombination coefficient	10^−11^ cm^3^s^−1^
*E*_ETL_	Energy of the ETL	3.8 eV
*E*_HTL_	Energy of the HTL	5.1 eV
*μ*_e,h_	Mobility of electrons and holes	Varied, but always *μ*_e_=*μ*_h_
		
*Selective layers*		
*d*_sel_	Thickness of selective layers	10^−6^ cm (10 nm)
*ɛ*_sel_	Dielectric permittivity of selective layers	10
*E*_G,sel_	Band gap energy of selective layers	See text
*E*_ETL_	Energy of the ETL	See text
*E*_HTL_	Energy of the HTL	See text
*N*_ETL (HTL)_	Effective density of states in the ETL (HTL)	5 × 10^20^ cm^−3^
*μ*_sel_	Mobility of electrons and holes	10 cm^2^(Vs)^−1^
*k*_sel_	Recombination coefficient	10^−15^ cm^3^ s^−1^
		
*Electrodes*		
*W*_F,e_	Work function of the electron contact	4.0 eV
*W*_F,h_	Work function of the hole contact	4.9 eV
*v*_e,h_	Surface recombination velocity of electrons and holes	10^12^ cm s^−1^

ETL, electron transport level; HTL, hole transport level.
