# Supplementary material for: Impact of charge transport on current–voltage characteristics and power-conversion efficiency of organic solar cells
Source: Nat Commun. 2015 Apr 24;6:6951. doi: 10.1038/ncomms7951 (PMC4421856; doi:10.1038/ncomms7951)
Supplement: Supplementary Information — Supplementary Figures 1-4 and Supplementary Note 1 [file ncomms7951-s1.pdf]

Supplementary Figures:

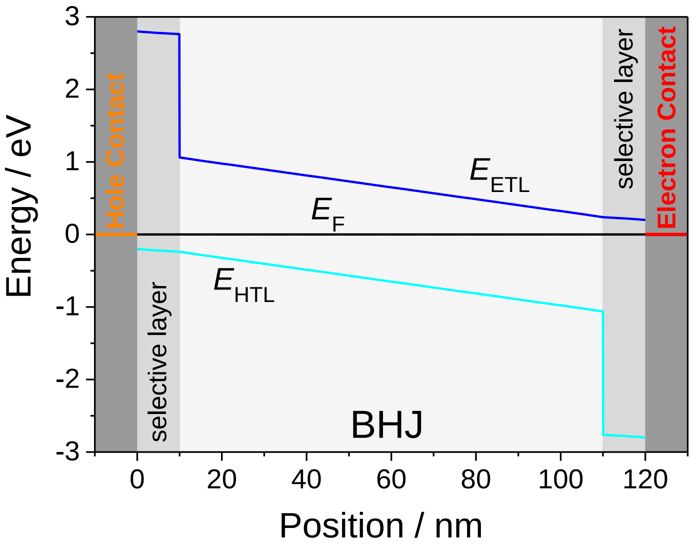

Supplementary Fig. 1: Energy level diagram in the dark. The plot shows the electron transport level (ETL) and the hole transport level (HTL) together with the Fermi energy as a function of the position for the effective semiconductor and the selective interlayers under dark and short-circuit conditions. The corresponding parameters can be found in Table 1.

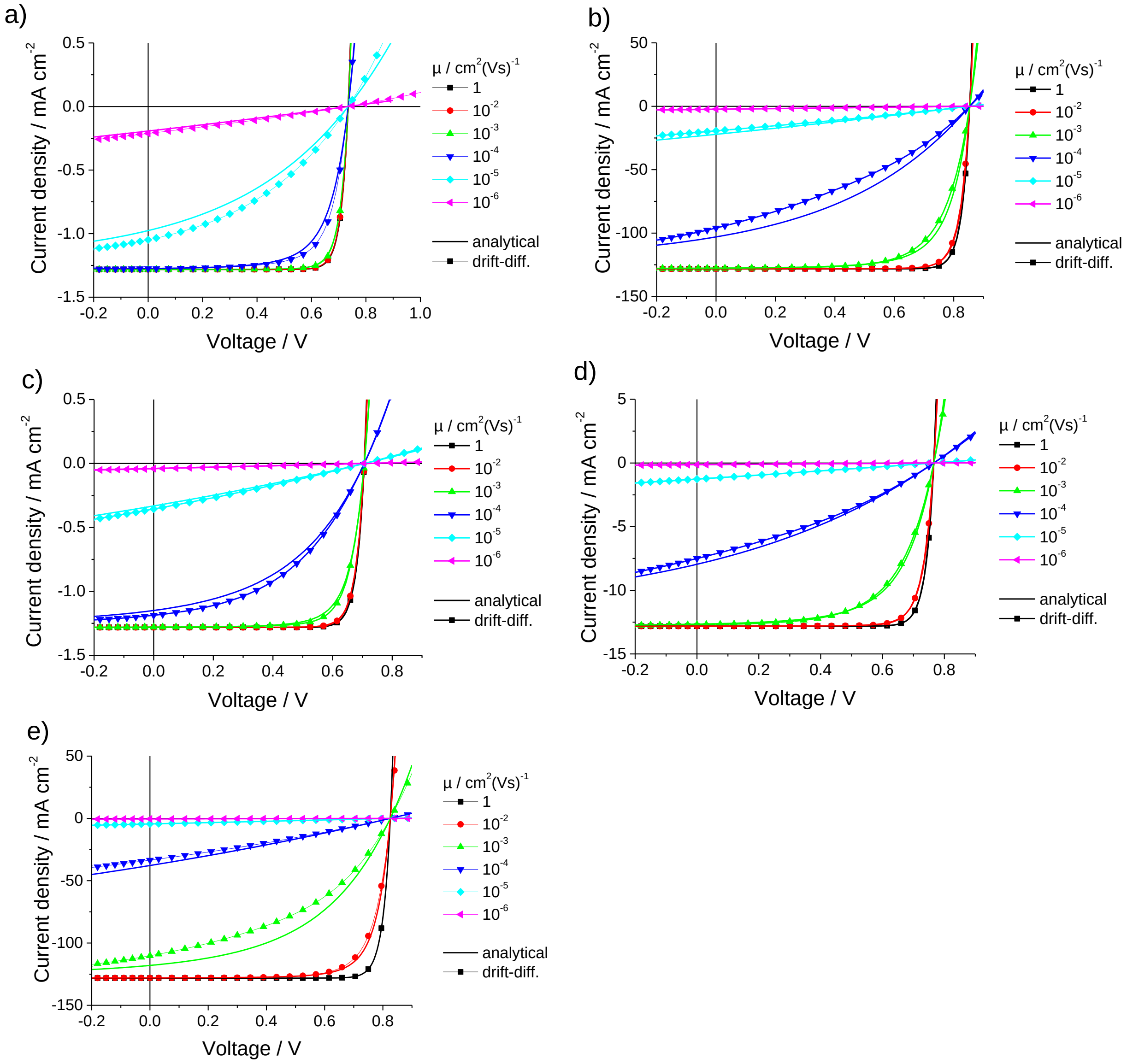

Supplementary Fig. 2: *JV*-characteristics under illumination for different mobilities and illumination intensities. *JV*-curves for six different mobilities under illumination intensities of 0.1, 1 and 10 suns and photoactive layer thicknesses of 100 and 300 nm, respectively. **a)** 100 nm, 0.1 sun; **b)** 100 nm, 10 suns; **c)** 300 nm, 0.1 sun; **d)** 300 nm, 1 sun; **e)** 300 nm, 10 suns. It gets clear that the effect of low mobilities on the *JV*-characteristics is more detrimental for higher illumination intensities and is even more pronounced if the layer thickness is increased to 300 nm.

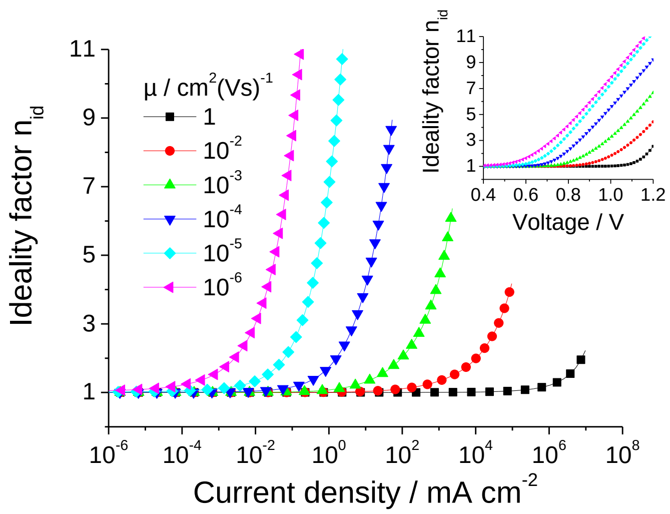

Supplementary Fig. 3: Determination of the ideality factor from dark *JV*-curves. The plot shows ideality factors under dark conditions as a function of current density or voltage (inset) for six different mobilities. The ideality factors were determined by the slope of the *JV*-curves in a log-linear plot. It can be seen that independent of mobility, the ideality factor is unity for negligible currents. It then starts to increase rapidly for increasing currents, this effect being more pronounced the lower the mobility. Therefore for practical measurements it is not feasible to obtain the correct value for n_{id} by fitting the *JV*-curve in the dark with the Shockley equation, except for (very) high mobilities.

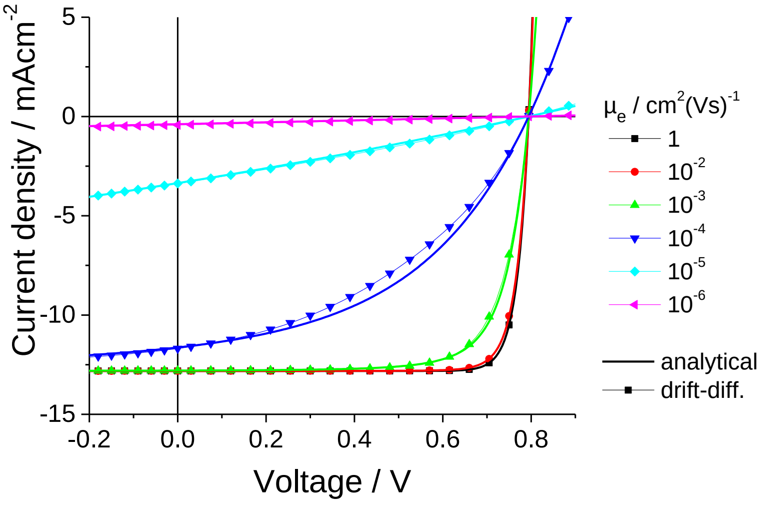

Supplementary Fig. 4: Unbalanced mobilities. *JV*-curves for an exemplary case of unbalanced mobilities. The same set of parameters was used as in Figure 1a, but with a threefold reduced hole mobility: $\mu_{\mathrm{e}} = 3\mu_{\mathrm{h}}$. For the analytical model, the effective mobility $\mu_{\mathrm{eff}} = \sqrt{\mu_{\mathrm{e}}\mu_{\mathrm{h}}}$ was used.

Supplementary Note 1:

We start with the condition, that in the bulk of the device, the current densities for electrons and holes, J_{e} and J_{h}, are the same. This is due to the fact, that both, generation and recombination are constant throughout the device. Under the assumption of identical gradients of E_{FE} and E_{FH}, we arrive at

$$\sigma_{\mathrm{e}} = \sigma_{\mathrm{h}} \rightarrow e n_{\mathrm{e}} \mu_{\mathrm{e}} = e n_{\mathrm{h}} \mu_{\mathrm{h}} \tag{1}$$

For unbalanced mobilities, the densities of electrons and holes will be different. For the case $\mu_{\mathrm{e}} > \mu_{\mathrm{h}}$ we can express this by

$$\begin{aligned} n_{\mathrm{e}} &= n - \Delta n \\ n_{\mathrm{h}} &= n + \Delta n \end{aligned} \tag{2}$$

with n being the average of the electron and hole densities. Combining (S2) with (S1) leads to:

$$\Delta n = \frac{\mu_\mathrm{e} - \mu_\mathrm{h}}{\mu_\mathrm{e} + \mu_\mathrm{h}} n$$

(3)

Then

$$\sigma = 2\sigma_\mathrm{e} = 2e n_\mathrm{e} \mu_\mathrm{e} = 2e(n + \Delta n)\mu_\mathrm{e} = 4en \frac{\mu_\mathrm{e}\mu_\mathrm{h}}{\mu_\mathrm{e} + \mu_\mathrm{h}} \quad (4)$$

Also

$$n_\mathrm{e} n_\mathrm{h} = n^2 - \Delta n^2 = \left[1 - \left(\frac{\mu_\mathrm{e} - \mu_\mathrm{h}}{\mu_\mathrm{e} + \mu_\mathrm{h}}\right)^2\right] n^2 \quad (5)$$

In the final step, expression (S5) is used to replace *n* in (S4), leading to

$$\sigma = 2e\sqrt{n_\mathrm{e} n_\mathrm{h} \mu_\mathrm{e} \mu_\mathrm{h}} = 2e\sqrt{n_\mathrm{e} n_\mathrm{h}}\, \mu_\mathrm{eff} \quad (6)$$

with the effective mobility $\mu_\mathrm{eff} = \sqrt{\mu_\mathrm{e}\mu_\mathrm{h}}$. Eq. (10) follows with $\sqrt{n_\mathrm{e} n_\mathrm{h}} = n_\mathrm{i} \exp\left[eV_\mathrm{int}/\left(2k_B T\right)\right]$.
